# Highly Sensitive Ultraviolet Photodetectors Fabricated from ZnO Quantum Dots/Carbon Nanodots Hybrid Films

**DOI:** 10.1038/srep07469

**Published:** 2014-12-12

**Authors:** Deng-Yang Guo, Chong-Xin Shan, Song-Nan Qu, De-Zhen Shen

**Affiliations:** 1 State Key Laboratory of Luminescence and Applications, Changchun Institute of Optics, Fine Mechanics and Physics, Chinese Academy of Sciences, Changchun 130033, China; 2 University of Chinese Academy of Sciences, Beijing 10049, China

## Abstract

Ultraviolet photodetectors have been fabricated from ZnO quantum dots/carbon nanodots hybrid films, and the introduction of carbon nanodots improves the performance of the photodetectors greatly. The photodetectors can be used to detect very weak ultraviolet signals (as low as 12 nW/cm^2^). The detectivity and noise equivalent power of the photodetector can reach 3.1 × 10^17^ cmHz^1/2^/W and 7.8 × 10^−20^ W, respectively, both of which are the best values ever reported for ZnO-based photodetectors. The mechanism for the high sensitivity of the photodetectors has been attributed to the enhanced carrier-separation at the ZnO/C interface.

Ultraviolet (UV) photodetectors have a variety of potential applications in both civilian and military areas including but not limited to flame sensing, UV radiation calibration and monitoring, astronomical studies, optical communication, missile launch detection, and so on^1^,^2^,^3^,^4^,^5^. Quantum dots (QDs) have some unique characters such as convenient integration, tunable bandgap, controllable transport and trap state properties^6^,^7^, which makes them an ideal candidate for photodetection^8^,^9^,^10^,^11^,^12^. Photodetectors fabricated from QDs have been studied extensively in recent years, and CdSe^13^, PbS^14^, HgTe^15^, PbSe^16^, ZnO^17^, etc., have been employed as the active layer of QD photodetectors. Amongst these materials, ZnO and its related materials have been considered as a promising candidate for UV photodetection due to their wide direct bandgap, high electron saturation velocity, high irradiation resistance, etc^17^,^18^,^19^. Additionally, one of the most noteworthy properties of ZnO lies in its rich and easy-to-get nanostructures^20^,^21^,^22^. ZnO QDs have been synthesized in various methods^21^,^22^, and UV photodetectors have been fabricated from these QDs^17^,^22^. Nevertheless, ZnO QDs usually have large surface-to-volume ratio and many grain boundaries, and the boundaries will hinder the mobility of electrons drastically thus deteriorate the performance of the photodetectors^23^,^24^,^25^. Therefore it is of great importance and significance if some method can be employed to enhance the performance of ZnO QD based photodetectors.

Carbon based nanostructures like graphene, nanotube, fullerene, etc has been employed to enhance the performance of photodetectors or photovoltaic devices^26^,^27^,^28^. Amongst these nanostructures, carbon nanodots have attracted much attention in recent years for their unique properties including highly luminescent characters, good photo-bleaching resistance, and mild synthesis method, etc^29^,^30^,^31^,^32^. Hence various potential applications have been expected from carbon nanodots, such as light-emitting devices, photocatalysis, bioimaging, and so on^29^,^30^,^31^,^32^,^33^,^34^. Besides, it has been reported that carbon nanodots can serve as an electron transportation layer^35^,^36^. It is speculated that if carbon nanodots are employed to facilitate the carrier transportation and separation of electrons and holes in ZnO QDs, the performance of ZnO QD photodetectors may be improved significantly. However, none such report can be found up to date.

In this work, carbon nanodots have been mixed together with ZnO QDs to form hybrid films, and UV photodetectors have been fabricated from the hybrid films. It is found that the responsivity of the photodetector can be increased greatly with the introduction of carbon nanodots. The detectivity and noise equivalent power (*NEP*) of the photodetector can reach 3.1 × 10^17^ cmHz^1/2^/W and 7.8 × 10^−20^ W, respectively, both of which correspond to the best values ever reported for ZnO-based photodetectors. The mechanism for the ultrahigh sensitivity has been attributed to the increase carrier separation at the ZnO/C interface.

## Results

**Figure 1:**
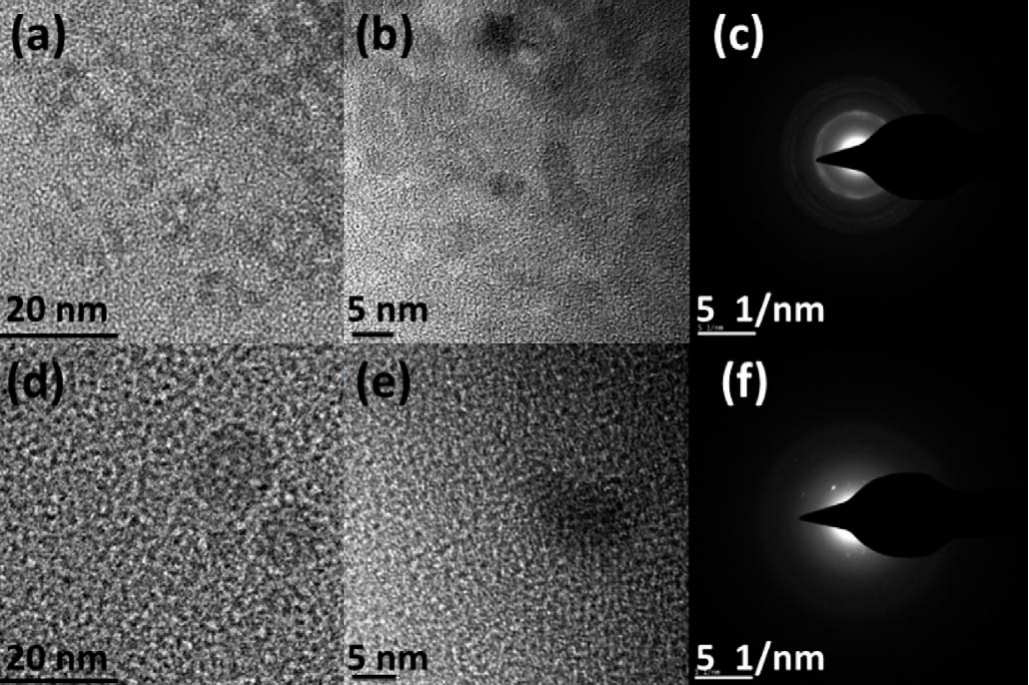
Low-magnification TEM image of the ZnO QDs (a) and carbon nanodots (d); High resolution TEM images of the ZnO QDs (b) and carbon nanodots (e); Selected-area electron diffraction patterns of the ZnO QDs (c) and carbon nanodots (f).

The morphology of the ZnO QDs and carbon nanodots are characterized by transmission electron microscopy (TEM), and the TEM images of the as-prepared ZnO QDs and carbon nanodots are shown in Fig. 1. Figures 1(a) and 1(d) show clearly that both the ZnO QDs and carbon nanodots have nearly spherical shape, and the size of the ZnO QDs is around 5 nm, while that of the carbon nanodots is about 20 nm. Figures 1(b) and 1(e) show the high-resolution TEM images of the ZnO QDs and carbon nanodots, respectively. Figures 1(c) and 1(f) indicate the corresponding selected area electron diffraction patterns of the two kinds of dots. The patterns exhibit lattice spacing and concentric rings, which indicates the crystalline nature of the QDs and nanodots.

**Figure 2:**
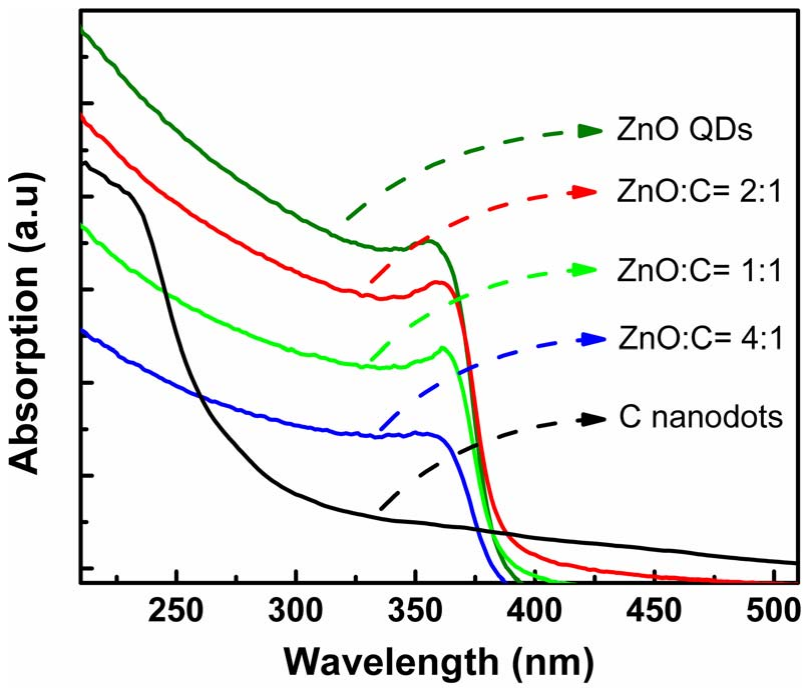
Absorption spectra of the ZnO QDs, carbon nanodots, and the blending solutions.

The typical absorption spectra of the ZnO QDs and carbon nanodots films with different volume ratios are shown in Fig. 2. Note that the spectra have been adjusted vertically for clarity. One can see from the spectra that the absorption curve of the hybrid films share the same lineshape with that of the ZnO QDs irrespective of the volume ratio. A strong absorption in UV region can be observed from the figure, while that in the visible region is negligible, which is favorable for high-performance UV photodetectors fabricated from the blending solution^37^.

**Figure 3:**
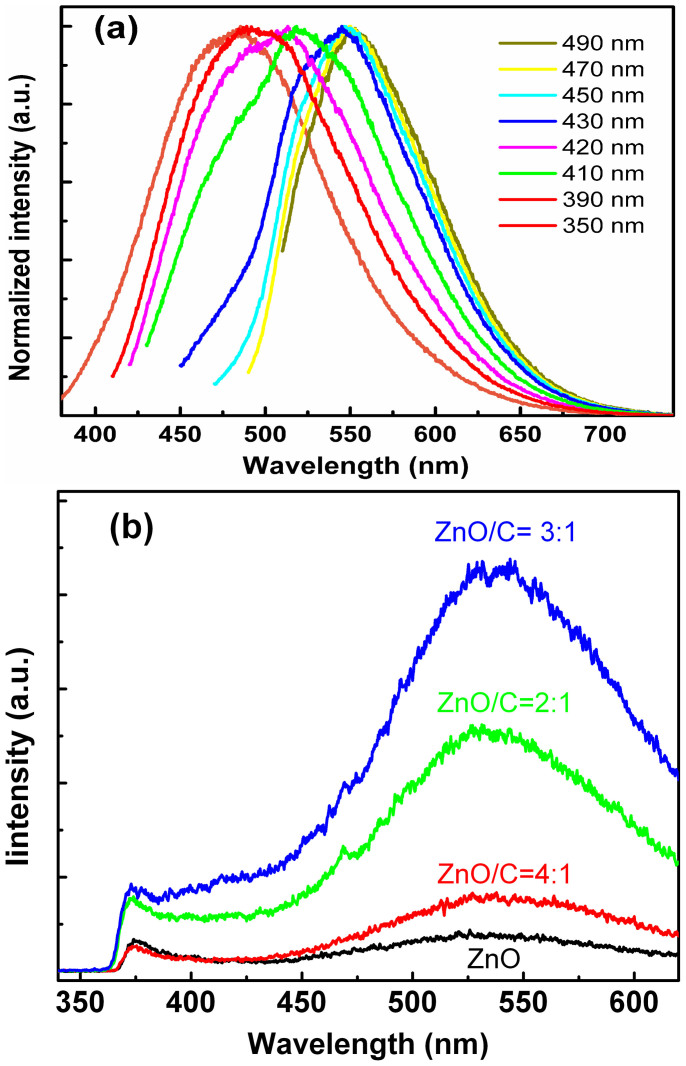
(a) Normalized PL spectra of the carbon nanodots excited by different wavelength illumination source; (b) PL spectra of ZnO/C blending solution with different volume ratios.

The photoluminescence (PL) spectra of the carbon nanodot solution are shown in Fig. 3. One can see from Fig. 3(a) that obvious emission can be detected from the carbon nanodots and the emission peak of the nanodots redshifts from around 480 nm to 550 nm when the excitation wavelength changes from 350 nm to 490 nm. Note that the above excitation dependent emission is a typical characteristic of carbon nanodots^29^,^30^,^31^. The PL spectra of the ZnO QD solution mixed with carbon nanodots with different volume ratio are shown in Fig. 3(b). All the spectra show a dominant broad emission at around 550 nm, which has been frequently observed in ZnO QDs, and can be attributed to the deep-level related emission of ZnO^38^,^39^. Another weak emission at around 375 nm is also visible from the figure, which can be attributed to the near band-edge emission of ZnO^39^. The PL spectra of the ZnO and carbon nanodots mixed solution share the same lineshape with that of ZnO QDs, but the emission intensity varies, which reveals that effective interaction between ZnO QDs and carbon nanodots occurs in the hybrid system.

**Figure 4:**
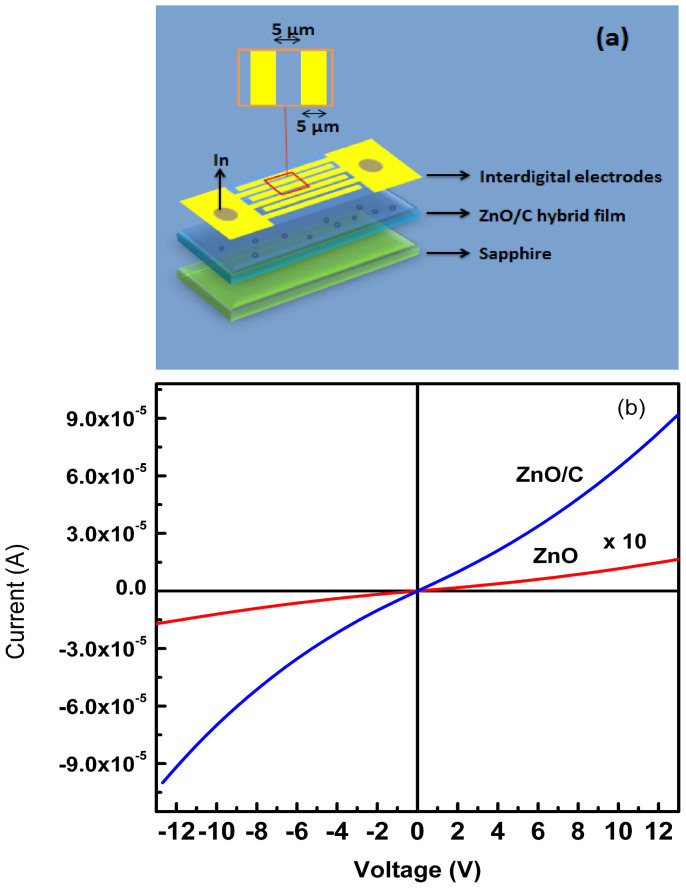
Schematic illustration (a) and I–V curves (b) of the photodetector fabricated from the ZnO/C hybrid films and bare ZnO QDs, note that the current of the device fabricated from bare ZnO QDs has been magnified by 10 times for clarity sake.

To fabricate the photodetectors, the mixed solution with different ZnO/C ratio was spin coated onto sapphire substrate, and then annealed in air ambient at 400°C for one hour, then at 600°C for another hour. In this way, ZnO QD/carbon nanodot hybrid films have been prepared, and the UV photodetectors have been fabricated from the hybrid films. The schematic diagram of the hybrid photodetector device structure fabricated from the ZnO QDs/carbon nanodots hybrid films is shown in Fig. 4(a). The electrode is composed by 12 pairs of interdigital contact, the length and width of the interdigital contacts are 500 μm and 5 μm, respectively, and the spacing between the adjacent interdigital contacts are 5 μm. Therefore the active area of the devices is around 5.75 × 10^−4^ cm^2^ (23 × 5 μm × 500 μm). Figure 4(b) shows the typical current-voltage (I–V) curves of the photodetector fabricated from the ZnO/C hybrid films with a ratio of 2:1 and bare ZnO QD film. One can see from the curves that in the investigated bias range, the current increases almost linearly with the bias voltage. Another noteworthy phenomenon lies in that the current of the hybrid photodetector is much larger than that of the bare ZnO QD photodetector, revealing that the introduction of carbon nanodots helps to decrease the resistivity of the ZnO QD films.

**Figure 5:**
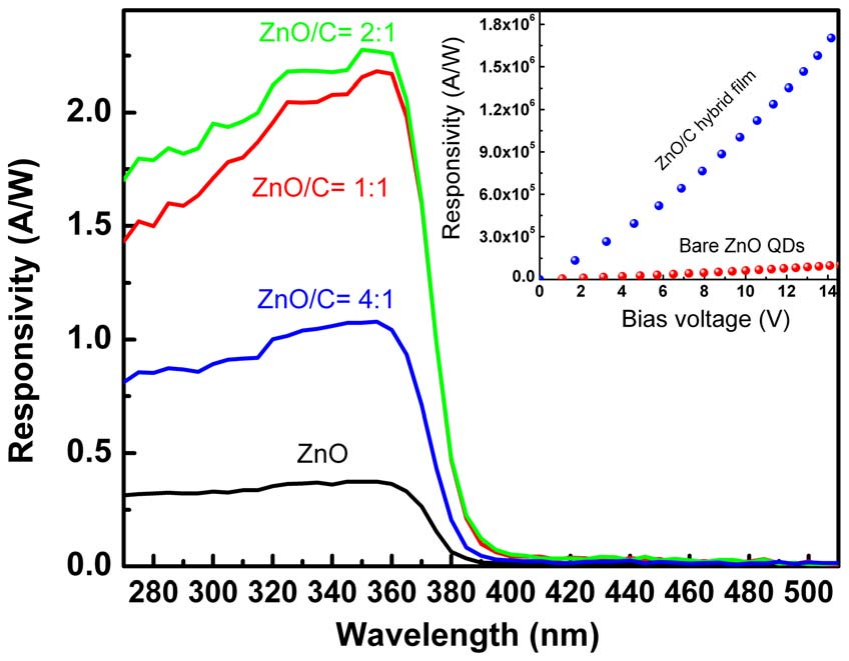
(a) Response spectra of the photodetectors fabricated from the ZnO QD/carbon nanodots hybrid film with different ratios under a bias of 50 mV; (b) Dependence of the responsivity of the photodetectors fabricated from the ZnO/C hybrid films and bare ZnO QDs on the bias voltage.

We note that the photodetectors show obvious response even when the illumination source is as weak as 12 nW/cm^2^. The response spectrum of the photodetectors fabricated from the hybrid films with different ratios at a bias voltage of 50 mV is shown in Fig. 5, and that of the photodetector fabricated from bare ZnO QDs is also plotted for comparison. One can see that the response spectra of the photodetector fabricated from the hybrid films are very similar in shape with that of the device fabricated from bare ZnO QDs, and all the spectra show a maximum response at around 370 nm. A noteworthy phenomenon in the spectra lies in the fact that the responsivity of the photodetectors fabricated from the hybrid films is much higher than that from bare ZnO QDs. Note that the response curves of the devices recorded one year later are almost identical to the ones recorded before as the devices are kept in a tinfoil sealed desiccator, indicating the good reliability of the photodetectors. The device fabricated from ZnO/C ratio of 2: 1 shows the largest responsivity, thus this device has been selected as a representative in the following investigations. The inset of Fig. 5 shows the dependence of the peak responsivity of the photodetectors fabricated from ZnO/C hybrid films and bare ZnO QDs on the bias applied. The responsivity increases monotonically with the bias in both cases, and the responsivity of the photodetector fabricated from ZnO QD/carbon nanodots hybrid films can reach 1.7 × 10^6^ A/W, while that of the photodetector fabricated from bare ZnO QDs is 9.7 × 10^4^ A/W reaches 14 V. That is, the responsivity of the device fabricated from the hybrid films is over two orders higher than that of the device fabricated from bare ZnO QDs.

**Figure 6:**
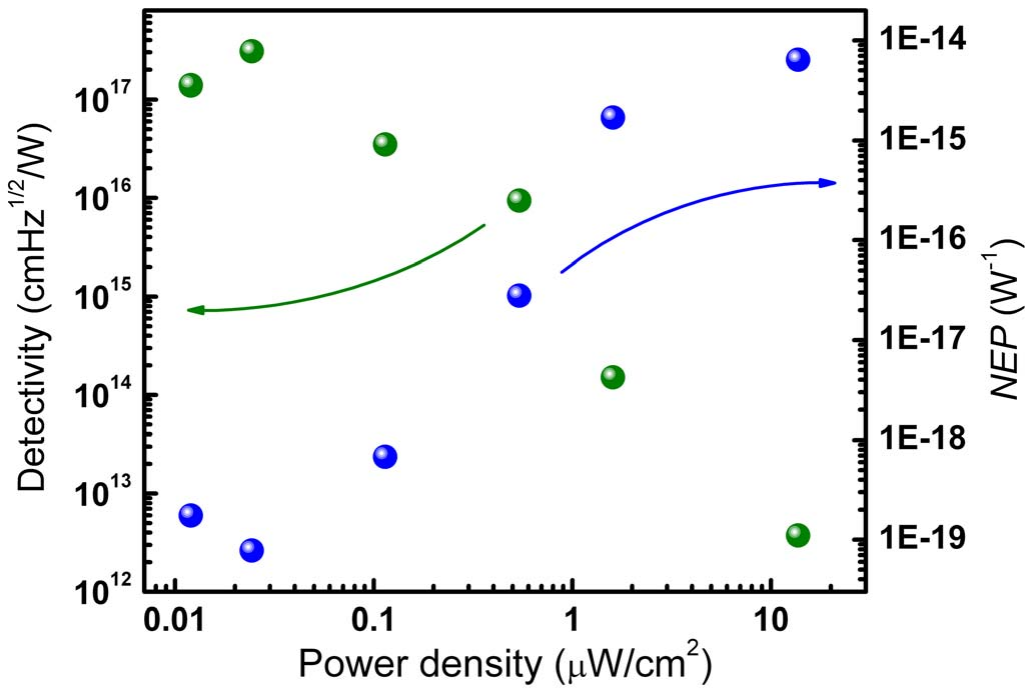
The dependence of the detectivity and *NEP* of the photodetectors fabricated from the hybrid film on the illumination power density.

Detectivity (D*) and noise equivalent power (*NEP*) are also two key figure-of-merit parameters that determine the performance of a photodetector, which usually correspond to the weakest signals that can be detected by the photodetector. D* ad *NEP* can be expressed by the following formulas^40^:


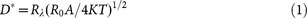



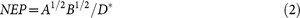
Where *R_λ_* is the responsivity of the devices; *R_0_* is the dark impedance; *A* is the detector area (5.75 × 10^−4^ cm^2^); *B* is the bandwidth. According to Eqs. 1 and 2, the detectivity and *NEP* of the photodetectors fabricated from the bare ZnO QDs and ZnO/C hybrid films have been measured. We note that the detectivity and *NEP* of the photodetector depends greatly on the power density of the illumination source, as shown in Fig. 6. One can see that the detectivity increases significantly with decreasing the illumination power density. The above fact reveals that the photodetectors in our case favors for weak signal detection, which is eagerly wanted in many practical application fields. When the power density of the illumination lamp is reduced to 20 nW/cm^2^, the detectivity of the photodetector fabricated from bare ZnO QDs can reach 7.8 × 10^16^ cmHz^1/2^/W, and the *NEP* can reach 3.1 × 10^−19^ W when the bias voltage is 14 V. While the corresponding values for the photodetector fabricated from the ZnO QD/carbon nanodots hybrid films are 3.1 × 10^17^ cmHz^1/2^/W and 7.8 × 10^−20^ W, respectively. Table I summarizes the recent reported relatively high detectivity and *NEP* values of ZnO-based UV photodetectors, and the corresponding values obtained in this work are also included. It is clear from the table that both the detectivity and *NEP* of the photodetectors fabricated from hybrid films correspond the best values ever reported for ZnO-based UV photodetectors^41^,^42^,^43^,^44^,^45^. Also note that the detectivity of the UV photodetectors fabricated from the hybrid films are significantly higher than that of conventional commercial available 4H-SiC UV photodetectors (~10^13^–10^15^ cmHz^1/2^/W)^46^,^47^.

## Discussion

**Figure 7:**
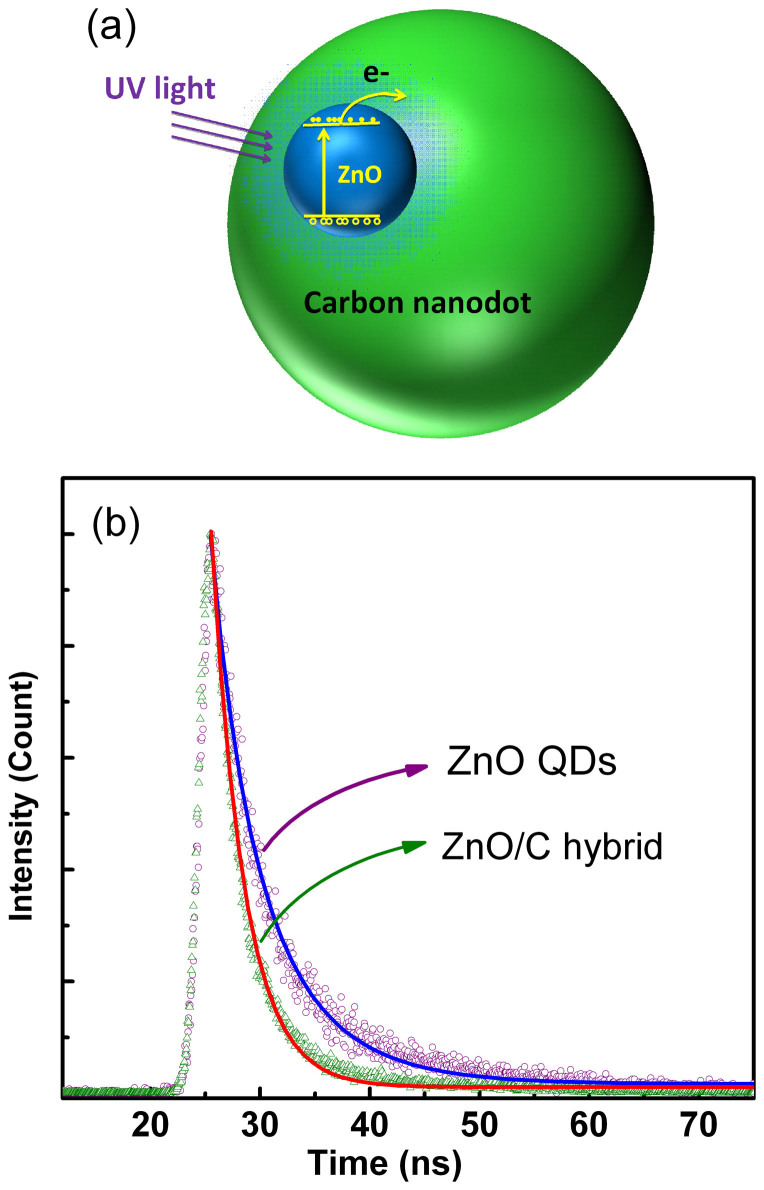
(a) Schematic illustration of the carrier generation and separation in the ZnO QDs/carbon nanodots hybrid structure; (b) Transient spectra of the emission at around 375 nm for bared ZnO QDs and ZnO/C hybrid films.

The above results indicate that the incorporation of carbon nanodots has improved the responsivity of the photodetectors greatly. In order to understand the mechanisms of the improved performance, a model has been proposed, as shown in Fig. 7(a). Since the absorption of the hybrid films is dominated by ZnO QDs, under the illumination of UV light, electrons and holes will be generated mainly in the ZnO QDs, and some of the generated electrons can enter into the carbon nanodots. Considering that carbon can serve as electron transportation media^32^,^44^, the electrons that entered into the carbon nanodots can be collected by the electrodes soon. To confirm this mechanism, the transient spectra of the emission at 375 nm for the hybrid ZnO/C system and bare ZnO QDs are illustrated in Fig. 7(b), in which the scattered symbols are experimental data, while the solid lines are fitting results to the experimental data using the following two-order exponential decay formula:
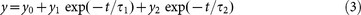


Where *y* is the emission intensity, *y_0_*, *y_1_*, *y_2_* are constant, *t* is time. One can see that in both cases, the experimental data can be well fitted, and the best fitting yields the lifetime of *τ*_1_ = 2.97 ns, and *τ*_2_ = 2.97 ns for the ZnO/C hybrid films, while *τ*_1_ = 6.28 ns, and *τ*_2_ = 3.16 ns for the bare ZnO QDs. Note that *τ*_1_ in both cases may come from the exciton recombination inside the ZnO QDs, while *τ*_2_ correspond to the exciton captured by the surface defects of the QDs^48^,^49^. It can be seen that the carrier lifetime for the ZnO/C hybrid films is distinctively shorter than that for the bare ZnO QDs, which consolidates that some of the carries have been transferred from the ZnO QDs to the carbon nanodots in the ZnO/C hybrid films, as illustrated in Fig. 7(a). The effective electron transportation from the ZnO QDs to the carbon nanodots will facilitate the carrier separation, thus helps to enhance the performance of the photodetector.

In summary, UV photodetectors have been fabricated from ZnO QDs/carbon nanodots hybrid films, and the detectivity and noise equivalent power of the photodetectors can reach 3.1 × 10^17^ cmHz^1/2^/W and 7.8 × 10^−20^ W, respectively, both of which corresponds to the best value ever reported for ZnO-based photodetectors. The mechanism for the high performance has been attributed to the increased carrier-separation at the ZnO QD/carbon nanodots interface. The results reported in this paper may provide a route to UV photodetectors with ultrahigh sensitivity that is eagerly wanted for weak signal detection.

## Methods

### Materials and synthesis

The ZnO QDs in this study were synthesized via a sol-gel method which involves with Zinc acetate dihydrate, 2-methoxyethanol and monoethanolamine (MEA)^50^. The synthesis process is illustrated as follows: Firstly, a three-neck flask loaded with 8.3 g zinc acetate dihydrate and a magnet rotor was installed with oil-bath and condenser pipe of which the straight part connecting with flask was sealed with nitrogen and the recycle part connecting with conduit was filled with water flow. After that 48 ml 2-methoxyethanol was added into the three-neck flask. The magnet rotor was kept rotary all the way. When the oil temperature was raised up to 70°C, 2.3 ml MEA was injected into the flask. After 24 hours stirring by rotary rotor, transparent and colourless ZnO QD solution was moved into an Erlenmeyer flask. The synthesis of carbon nanodots was carried out according to the method developed in Ref. 32, in which octadecylene (ODE), 1-hexadecylamine (HDA) and anhydrous citric acid were engaged in the synthesis. Firstly, 15 ml ODE acting as inert solvent and 1.5 g HAD acting as surface passivation agent were loaded in a three-neck flask, and then heated to boiling under argon flow. Secondly, 1.0 g anhydrous citric acid acting as carbon precursor was injected into the flask quickly. After purified 5 times by acetone, jelly-like carbon nanodots were obtained, and the product was then dissolved into toluene.

### Device fabrication and characterization

To prepare ZnO/carbon nanodot hybrid films, the ZnO QDs and carbon nanodots solution were mixed in a series of volume ratio (1:0, 4:1, 2:1, 1:1), and the mixed solution was spun onto *c*-plane sapphire and then the samples were annealed at 400°C for one hour, and then at 600°C for another hour. In this way, ZnO QD and carbon nanodot hybrid films have been prepared. To fabricate UV photodetectors from the hybrid films, a thin Au layer was deposited onto the hybrid films in a vacuum evaporation method acting as contact, and interdigital electrodes were configured via a photolithography and wet etching process. For comparison, ZnO layers without the carbon nanodots have also been prepared, and photodetectors have been fabricated from the ZnO layers. The morphology of the QDs was characterized using a Philips TF-F20 transmission electron microscope operating at 200 kV. The absorption spectra of the ZnO QDs and carbon nanodots were studied in a Shimadzu UV-3101 PC scanning spectrophotometer. The photoluminescence spectra of the carbon nanodots and ZnO QDs are measured in a Shimadzu F4500 spectrometer with a Xe lamp as the excitation source. Photoresponse properties of the hybrid films were measured in a SPEX scanning monochromator employing a 150 W Xe lamp as the illumination source. The current- voltage measurement of the photodetector was measured in a Lakeshore 7707 Hall system.

## Author Contributions

D.Y.G. conducted the measurements of the ZnO QDs and carbon nanodots, and also the response properties of the photodetectors, C.X.S. conceive the idea of the paper, and analyze the experimental data. S.N.Q. took part in the discussion and formation of the idea of this paper, D.Z.S. is the principle investigator of the project.

## Figures and Tables

**Table 1:** Comparison of the D* and *NEP* of the photodetectors fabricated from the ZnO QDs/carbon nanodot hybrid films with the reported values

ZnO structure	D* (cmHz^1/2^/W)	*NEP* (W)	Voltage (V)	Reference
Thin film	1.19 × 10^10^	2.65 × 10^−12^	5	41
Nanowall	3.38 × 10^9^	1.87 × 10^−10^	2	42
Nanorod	1.43 × 10^15^	2.27 × 10^−14^	5	43
Nanowire	2.13 × 10^9^	5.88 × 10^−13^	1.5	44
Nanoparticle	3.43 × 10^15^	NA	9	45
ZnO/C Hybrid film	3.1 × 10^17^	7.8 × 10^−20^	14	This work
